# Current Trends in Food-Derived Peptidic Antioxidants

**DOI:** 10.3390/antiox11050962

**Published:** 2022-05-12

**Authors:** Henry M. Corpuz, Soichiro Nakamura, Shigeru Katayama

**Affiliations:** 1 Rice Chemistry and Food Science Division, Philippine Rice Research Institute, Science City of Muñoz 3119, Nueva Ecija, Philippines; hmcorpuz@exchange.philrice.gov.ph; 2 Department of Bioscience and Biotechnology, Shinshu University, 8304 Minamiminowamura, Nagano 399-4598, Japan

Recently, peptidic antioxidants have attracted much attention due to their promising applications in the production of valuable functional food and nutraceuticals with health-promoting properties. Food-derived peptidic antioxidants produced by enzymatic hydrolysis, simulated gastrointestinal digestion, microbial fermentation, and other food-processing technologies provide nutritional and biological properties, including the prevention of foods’ quality deterioration and the management of chronic non-communicable diseases (NCDs). Diabetes is a serious health problem worldwide. In 2021, it was estimated that 537 million adults aged 20–79 years have diabetes and that 6.7 million people died from the consequences of high blood sugar (IDF, 2021). The increasing incidence of diabetes is linked to sedentary lifestyles and unhealthy diets. Dietary intervention through the consumption of functional foods may have a significant impact on managing blood sugar levels and increasing insulin sensitivity. Cereal-derived bioactive peptides and protein hydrolysates with antidiabetic properties have been reported. Peptide hydrolysates obtained from oat bran with good radical scavenging activities showed antidiabetic activity in vitro by inhibiting α-amylase and dipeptidyl peptidase-4 enzymes and enhancing the secretion of glucagon-like peptide-1 in NCI-H716 cells [1].

Industrial by-products containing high-quality proteins are being used to produce cheap antioxidant peptides with promising therapeutic and nutritional applications. For instance, despite being a by-product of the egg industry, the muscle of spent laying hens that reach the end of the egg-laying cycle is an excellent source of meat proteins for low-cost antioxidant peptides with antihypertensive effects. Reactive oxygen species (ROS) play critical roles in the homeostasis of the vascular wall; hence, they are involved in the development and progression of cardiovascular diseases. Given the adverse outcomes of vascular oxidative stress in hypertension, Fan et al. [2] evaluated the antioxidant effects of spent hen-derived peptides in vascular smooth muscle A7r5 cells (VSMCs) and endothelial EA.hy926 cells (ECs). The antioxidant effect of VRP, LKY, and VRY (angiotensin-converting enzyme (ACE) inhibitors peptides) is due to their ROS-scavenging activity. Meanwhile, VVHPKESF (ACE2 upregulator peptide) attenuated oxidative stress in VSMCs by upregulating endogenous antioxidant enzymes glutathione peroxidase 4 and superoxide dismutase (SOD) 2 via ACE2– MasR pathway.

The production of antioxidant peptides involves numerous steps. In the preparation and hydrolysis of protein extracts, choosing a suitable pH and temperature is necessary to improve the yield without affecting the bioactivities and functional properties of the target peptides. Rao et al. [3] investigated the effect of pH and temperature in modulating the release of peptides with antioxidant activity after simulating in vitro gastrointestinal digestion of egg white proteins (EWP). They found that heat treatment at 60 °C at a neutral pH can improve the digestibility of EWP and could potentially enhance the release of antioxidant peptides.

Protein and peptide modifications through bioconjugations (e.g., glycosylation, phosphorylation, and lipophilization) are being employed to design new bioactive compounds with improved functionalities. Conjugating the proteins with carbohydrate polymers significantly increased their reducing power and antioxidant activity, as observed in dextran-conjugated soy proteins and galactomannan-conjugated ovalbumin, lysozymes, and phosvitin [4]. These bioconjugated proteins and peptide fractions could be used as value-added functional ingredients for food systems that require antioxidant protection. Zhang et al. [4] assessed the potential improvement of antioxidant activity of casein phosphopeptide (CPP) conjugated with galactomannan or xyloglucan. Consequently, the addition of galactomannan or xyloglucan to CPP using Maillard reaction conditions significantly reduced its bioactivity in the Fenton reaction-deoxyribose assays, with no adverse effects on its antioxidant activity in both ABTS and liposome assays [4]. An antimicrobial peptide (MS15) with a molecular mass of 6091 Da produced by *Bacillus velezensis* isolated from Kimchi displayed significant antioxidant properties in vitro and in a cell model. Peptide MS15 exhibited excellent ABTS^•+^, DPPH, and SOD radical-scavenging activity and electron-donating capacity using FRAP and CUPRAC assays in a dose-dependent manner. Pre-treatment with MS15 peptides also suppressed the ROS formation in RAW264.7 cells by modulating the nuclear factor erythroid 2-related factor-2 (Nrf2)-heme oxygenase-1(HO-1) signaling pathway [5].

The review articles in this Special Issue discuss the emerging technologies and recent trends in the preparation, purification, characterization, and evaluation of the potential health benefits of antioxidant peptides isolated from foods source such as soybean, wheat, corn, rice, nuts, seeds, collagen, milk, eggs, animal muscle protein, fish, and food-processing by-products [6]. In addition, Matemu et al. [7] presented antioxidant peptides from legumes with a high amino acid score, namely soybean, chickpea, lentil, cowpea, and mung bean, and their roles in the prevention of oxidative-stress-induced chronic illnesses such as hypertension, cancers, and inflammatory diseases. They also highlighted the latest advances in antioxidant peptides preparation, purification, identification, and bioassays. Finally, Ofosu et al. [8] reviewed the current production techniques for cereal antioxidant peptides and their plausible mechanism of action in reducing and managing the risks of metabolic syndrome such as type 2 diabetes, obesity, hypertension, cardiovascular diseases, and some cancers.

In summary, food-derived antioxidant peptides could be developed as health-promoting functional foods or ingredients and dietary supplements for the prevention and management of NCDs and metabolic disorders induced by oxidative stress.
